# Effect of Glycan Shift on Antibodies against Hepatitis C Virus E2 412–425 Epitope Elicited by Chimeric sHBsAg-Based Virus-Like Particles

**DOI:** 10.1128/spectrum.02546-22

**Published:** 2023-01-31

**Authors:** Anna Czarnota, Anna Offersgaard, Ania Owsianka, Garazi Peña Alzua, Jens Bukh, Judith Margarete Gottwein, Arvind H. Patel, Krystyna Bieńkowska-Szewczyk, Katarzyna Grzyb

**Affiliations:** a Laboratory of Virus Molecular Biology, Intercollegiate Faculty of Biotechnology of the University of Gdańsk and Medical University of Gdańsk, Gdańsk, Poland; b Copenhagen Hepatitis C Program (CO-HEP), Department of Infectious Diseases, Copenhagen University Hospital—Hvidovre, Hvidovre, Denmark; c Department of Immunology and Microbiology, Faculty of Health and Medical Sciences, University of Copenhagen, Copenhagen, Denmark; d MRC-University of Glasgow Centre for Virus Research, Glasgow, United Kingdom; Indian Institute of Science Bangalore

**Keywords:** glycan shift, hepatitis B virus, hepatitis C virus, vaccines, virus-like particles

## Abstract

Two of the most important mechanisms of hepatitis C virus (HCV) immune evasion are the high variability of the amino acid sequence and epitope shielding via heavy glycosylation of the envelope (E) proteins. Previously, we showed that chimeric sHBsAg (hepatitis B virus [HBV] small surface antigen)-based virus-like particles (VLPs) carrying highly conserved epitope I from the HCV E2 glycoprotein (sHBsAg_412–425) elicit broadly neutralizing antibodies (bnAbs). However, many reports have identified escape mutations for such bnAbs that shift the N-glycosylation site from N417 to N415. This shift effectively masks the recognition of epitope I by antibodies raised against the wild-type glycoprotein. To investigate if glycan-shift-mediated immune evasion could be overcome by targeted vaccination strategies, we designed sHBsAg-based VLPs carrying epitope I with an N417S change (sHBsAg_N417S). Studies in BALB/c mice revealed that both sHBsAg_412–425 and sHBsAg_N417S VLPs were immunogenic, eliciting antibodies that recognized peptides encompassing epitope I regardless of the N417S change. However, we observed substantial differences in E1E2 glycoprotein binding and cell culture-derived HCV (HCVcc) neutralization between the sera elicited by sHBsAg_412–425 and those elicited by sHBsAg_N417S VLPs. Our results suggest a complex interplay among antibodies targeting epitope I, the E1E2 glycosylation status, and the epitope or global E1E2 conformation. Additionally, we observed striking similarities in the E1E2 glycoprotein binding patterns and HCVcc neutralization between sHBsAg_412–425 sera and AP33, suggesting that the immunization of mice with sHBsAg_412–425 VLPs can elicit AP33-like antibodies. This study emphasizes the role of antibodies against epitope I and represents an initial effort toward designing an antigen that elicits an immune response against epitope I with a glycan shift change.

**IMPORTANCE** Epitope I, located within amino acids 412 to 423 of the HCV E2 glycoprotein, is an important target for an epitope-based HCV vaccine. One interesting feature of epitope I is the N417 glycosylation site, where a single change to S417 or T417 can shift the glycosylation site to position N415. This shift can effectively prevent the binding of broadly neutralizing antibodies targeting epitope I. Aiming to overcome glycan-shift-mediated immune evasion, we constructed sHBsAg_N417S VLPs carrying E2 epitope I, with N417S, and compared them with VLPs carrying wild-type epitope I. We show that antibodies elicited by the sHBsAg-based VLPs presenting two variants of the 412–425 epitope targeted two distinct glycan variants of the HCV E1E2 heterodimer. Our study suggests that due to the conformational flexibility of the E2 glycoprotein and epitope I, future vaccine antigens should elicit antibodies targeting more than one conformation and glycosylation variant of the 412–423 epitope.

## INTRODUCTION

It is estimated that at least 58 million individuals worldwide are chronically infected with hepatitis C virus (HCV), resulting in increased risks of chronic liver disease and hepatocellular carcinoma, causing about 300,000 deaths, annually (1). In recent years, new antiviral therapies based on direct-acting antivirals (DAAs) have been introduced (2). However, due to poor diagnostic coverage and high treatment costs, it has become apparent that in many countries, only a small fraction of HCV-infected individuals will receive DAA therapy. In addition, reinfections among people who inject drugs are common, and emerging resistance to DAAs is expected to compromise treatment efficacy (3, 4). Thus, the development of an effective prophylactic vaccine against HCV is required to control the spread of this virus (5, 6) and to support disease elimination efforts. Such a vaccine should ideally generate broadly neutralizing antibodies (bnAbs) able to prevent infection caused by various circulating genotypes and subtypes of HCV. However, the high genetic heterogeneity and conformational flexibility of the envelope glycoproteins of HCV pose a major obstacle to effective vaccine design (5, 7).

The prime targets of host neutralizing antibodies, the HCV envelope glycoproteins E1 and E2, located on the surface of the viral particle, show extensive genetic heterogeneity, particularly in the hypervariable regions localized in the E2 glycoprotein (8, 9). Thus, an effective vaccine should preferentially raise neutralizing antibodies against genetically conserved regions of the E1E2 heterodimer to elicit bnAbs (7, 10, 11).

The most important linear neutralizing HCV epitope and, thus, an important vaccine target is the antigenic domain comprising epitope I located within amino acids (aa) 412 to 423 of the E2 glycoprotein (412–423 epitope). This region is involved in HCV binding to the CD81 entry receptor (12, 13) and is conserved among over 5,500 E2 sequences in the GenBank database (14). However, Tarr et al. highlighted the low immunogenicity of the 412–423 epitope in natural infection, as the prevalence of antibodies against epitope I in sera from HCV-infected patients was only 2.5% (15). Therefore, the efficient presentation of the 412–423 epitope to the immune system should be an important goal for HCV vaccine design. We previously showed that the expression of amino acids 412 to 425, encompassing the 412–423 epitope, on the hepatitis B virus (HBV) small surface antigen (sHBsAg) elicited high levels of bnAbs against HCV (16).

One interesting feature of epitope I is the N417 glycosylation site, defined by the canonical N-X-S/T glycosylation signal sequence, where a single change to S417 or T417 can shift the glycosylation site to position N415 (17). Several monoclonal antibodies (mAbs) targeting epitope I have been isolated from rodents and humans, including mouse AP33 (12, 14, 18), rat 3/11 (18, 19), human HCV1 (20) and HC33.1 (21), as well as humanized MRCT10.v362 and hu5B3.v3 (17). However, the N-linked glycan shift can effectively prevent the binding of the broadly neutralizing AP33, the humanized counterparts, and HCV1 to epitope I (14, 17). Furthermore, HCV escape variants carrying N417S/T glycan shift changes were selected during treatment with mAbs targeting epitope I (22, 23).

To investigate if glycan-shift-mediated immune evasion could be overcome by targeted vaccination strategies, we constructed sHBsAg_N417S virus-like particles (VLPs) carrying E2 aa 412 to 425, comprising epitope I, with the N417S change (QLINT**S**GSWHINST [change indicated in boldface type]), thereby introducing a glycan shift into the epitope. These particles were compared with previously described sHBsAg_412–425 VLPs presenting the wild-type 412–425 epitope (QLINT**N**GSWHINST) on the surface of sHBsAg VLPs (24). In this study, we compared the binding of antibodies elicited by the sHBsAg_N417S and sHBsAg_412–425 VLPs to HCV peptides and native HCV E1E2 complexes with and without the N417S change and tested the impact of the N417S change on the neutralization of cell culture-infectious HCV (HCVcc). Moreover, we compared the E1E2 binding pattern of the antibodies elicited by the sHBsAg-based VLPs to that of well-characterized, broadly neutralizing mAbs.

## RESULTS

### Expression and characterization of chimeric sHBsAg-based particles.

**FIG 1:**
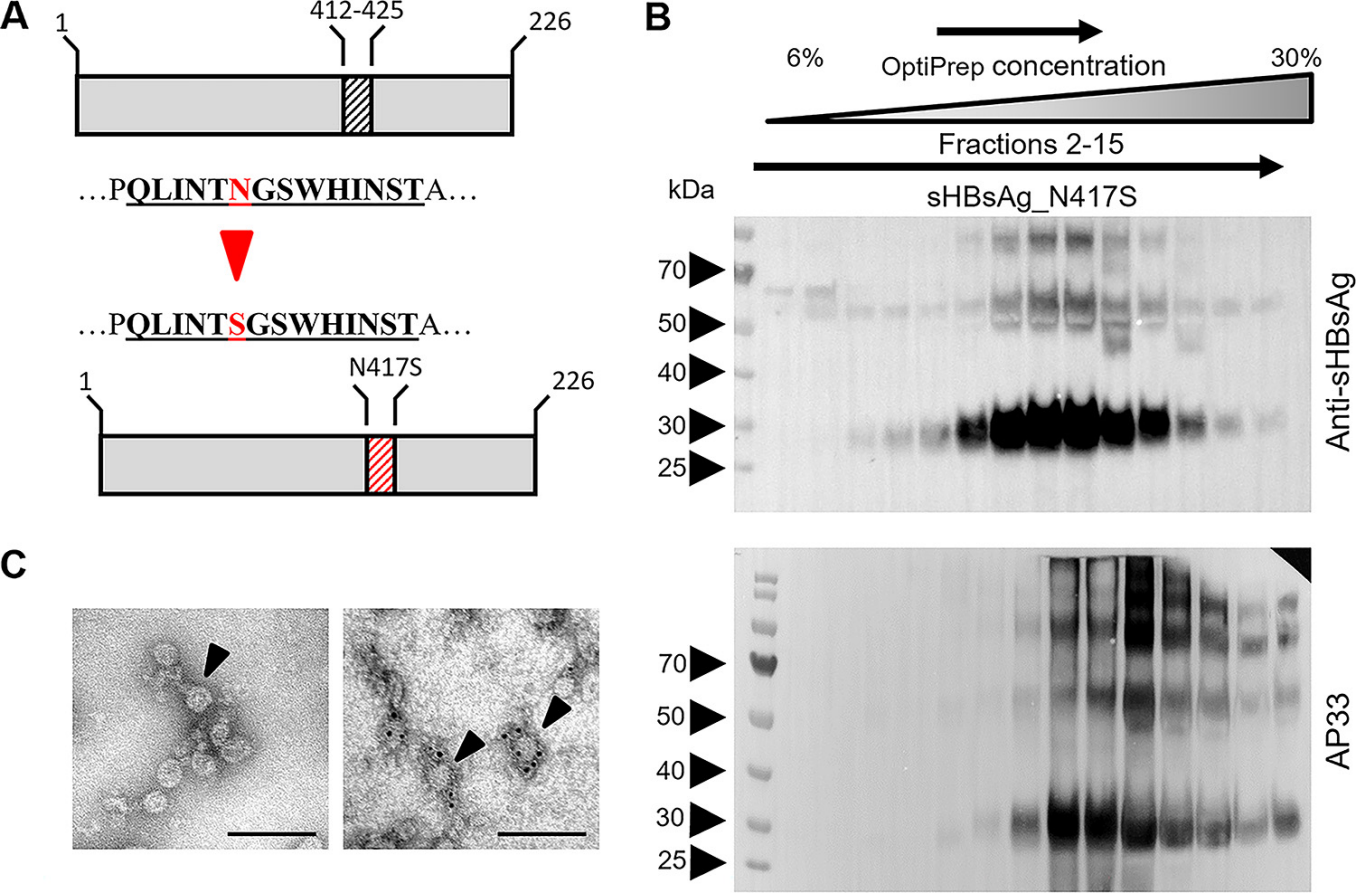
Construction of chimeric proteins. (A) The 412–425 sequence was derived from HCV isolate H77C (GenBank accession no. AF011751) and comprises epitope I (aa 412 to 423) with two additional amino acids, S424 and T425. The N417S epitope was generated by an N417 substitution with S in the 412–425 epitope; changed amino acids are marked in red. Chimeric constructs were generated by the insertion of the sequences coding for HCV E2 epitopes into the sequence coding for the hydrophilic loop of the sHBsAg protein at position P127/A128. (B) To concentrate and partially purify the chimeric particles, lysates from L. tarentolae cell cultures expressing chimeric proteins were placed on top of an OptiPrep density gradient. Seventeen fractions of 0.5 mL were harvested from top to bottom. Aliquots of fractions 2 to 15 were then analyzed using Western blotting with anti-sHBsAg pAb or AP33. On the left protein ladder, the molecular weight in kilodaltons is given. (C) Electron micrographs of chimeric sHBsAg-based particles. (Left) After concentration on the OptiPrep density gradient, the chimeric particles were stained with uranyl acetate and analyzed using electron microscopy. The observed particles were approximately 20 to 30 nm in diameter. (Right) The binding of AP33 to the N417S epitope was studied by immunogold labeling using secondary goat anti-mouse antibodies conjugated with 6-nm gold particles. Black arrowheads indicate chimeric VLPs. Bars, 100 nm.

The construction, expression, and characterization of the sHBsAg_412–425 VLPs were described previously (16, 24). In short, the sequence coding for the HCV (genotype 1a isolate H77C [25]) E2 glycoprotein region spanning residues 412 to 425 was inserted into the sequence of the major antigenic loop of the HBV sHBsAg protein at positions corresponding to amino acid P127/A128. The addition of amino acids S424 and T425 to epitope I (aa 412 to 423) retained the natural N2 glycosylation site at N423. For the generation of sHBsAg_N417S VLPs, a single point mutation was introduced into the sequence coding for the 412–425 epitope, changing asparagine (N417) to serine (S417) (Fig. 1A).

Chimeric VLPs were produced in high-density cell cultures using the tetracycline-inducible Leishmania tarentolae expression system and in mammalian HEK293F cells. The sHBsAg_N417S VLPs were concentrated and partially purified from the cell lysate by ultracentrifugation on an OptiPrep gradient. The expression of proteins was confirmed by SDS-PAGE of the gradient fractions followed by Western blotting with sHBsAg-specific antibodies. The molecular mass of the sHBsAg_N417S monomer was approximately 30 kDa, and most of the sHBsAg_N417S protein was distributed in fractions 8 to 11 (Fig. 1B). Particle assembly was confirmed by transmission electron microscopy. The sHBsAg-positive fractions showed spherical particles approximately 25 nm in diameter (Fig. 1C, left). Additionally, immunogold labeling of the sHBsAg_N417S particles with AP33 revealed the selective labeling of the VLPs, strongly suggesting that despite the N417S change, the presented epitope was successfully recognized by AP33 (Fig. 1B and C, right; see also Fig. S1A in the supplemental material).

To test the glycosylation status of the L. tarentolae-derived VLPs, we treated them with peptide *N*-glycosidase F (PNGase F) and endo-β-*N*-acetylglucosaminidase Hf (endo Hf) (Fig. S1B). We observed a shift in the migration patterns for the samples treated with PNGase F; however, they were unaffected by endo Hf. This suggests that VLPs produced in the L. tarentolae system are composed of complex and not hybrid or high-mannose glycans. Similar results were observed for VLPs produced in human embryonic kidney HEK293F cells (Fig. S1B).

**FIG 2:**
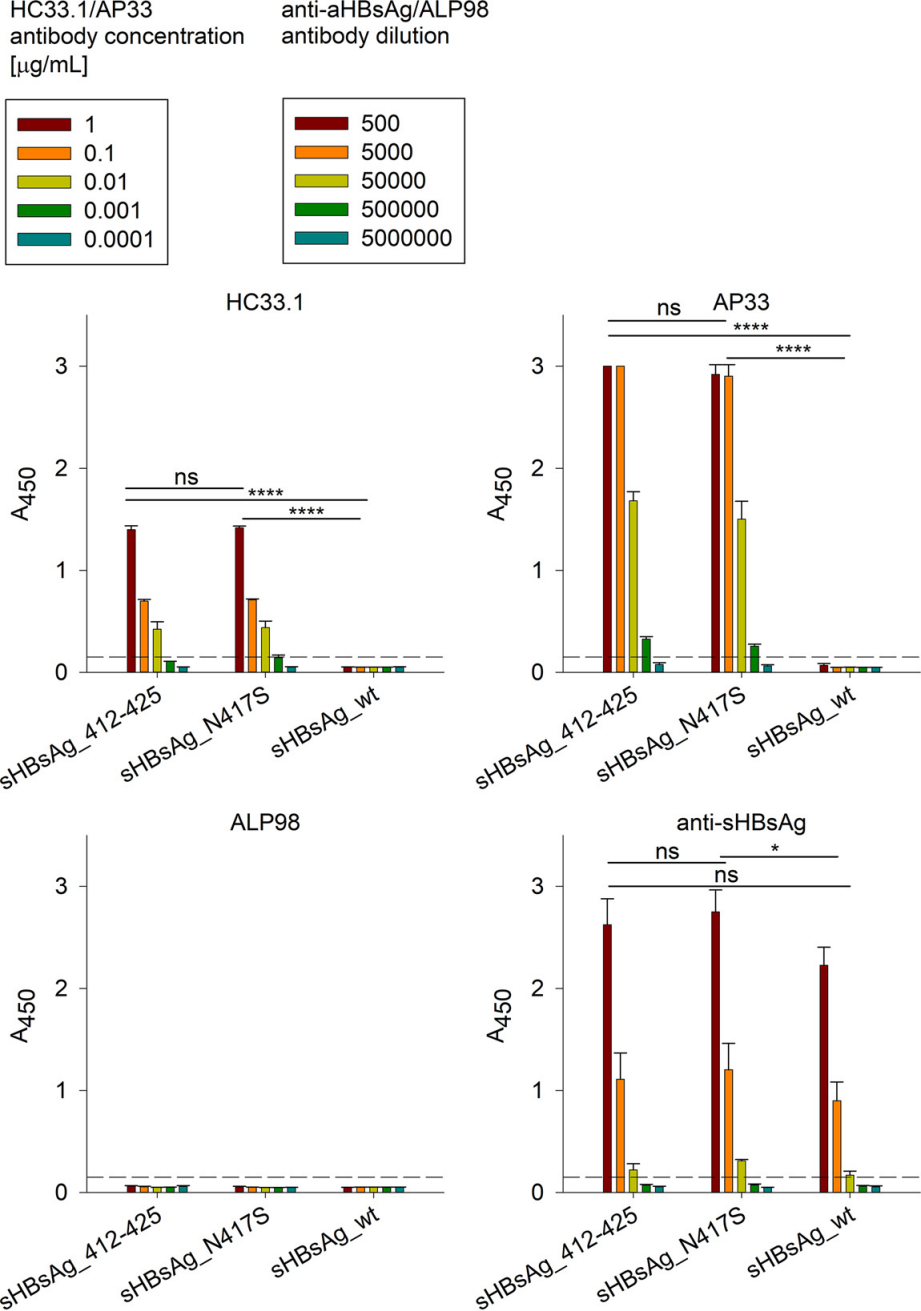
Recognition of VLPs by a panel of antibodies. ELISA plates were coated with partially purified sHBsAg_412–425, sHBsAg_N417S, and sHBsAg_wt VLPs, as depicted on the *x* axis. VLPs were probed with serial dilutions of epitope I-specific AP33/HC33.1 and anti-sHBsAg pAb. mAb ALP98 was used as a negative control. Data were analyzed using two-way analysis of variance (ANOVA) (ns [not significant], *P* > 0.05; *, *P* < 0.05; ****, *P* < 0.0001). The mean *A*_450_ values are shown on the *y* axis. The data represent the results from two independent experiments performed in duplicate, and error bars indicate standard deviations.

To further characterize sHBsAg_N417S VLPs, we used the following HCV E2 412–423 epitope-specific antibodies: AP33, which is unable to recognize the E2 glycoprotein carrying the N417S change (17, 26), and HC33.1, which binds E2 with the N-glycosylation site at either N417 or N415, with a higher affinity for the N415 variant (27). Interestingly, sHBsAg_N417S VLPs were recognized by AP33 with the same efficiency as that for sHBsAg_412–425 VLPs in an enzyme-linked immunosorbent assay (ELISA), with no cross-reactivity with sHBsAg particles (Fig. 2). Similarly, HC33.1 bound both sHBsAg_412–425 and sHBsAg_N417S particles, with no differences in affinity being observed between the two (Fig. 2). Similar results were obtained for sHBsAg_412–425 and sHBsAg_N417S VLPs expressed in mammalian HEK293F cells (Fig. S2).

In summary, we were able to confirm sHBsAg_N417S chimeric protein production and VLP formation. Additionally, we observed that AP33, which was sensitive to the E2 N417S change, unexpectedly bound to sHBsAg_N417S VLPs and sHBsAg_412–425 VLPs with similar efficiencies.

### Immunogenicity of the chimeric sHBsAg-based particles.

**FIG 3:**
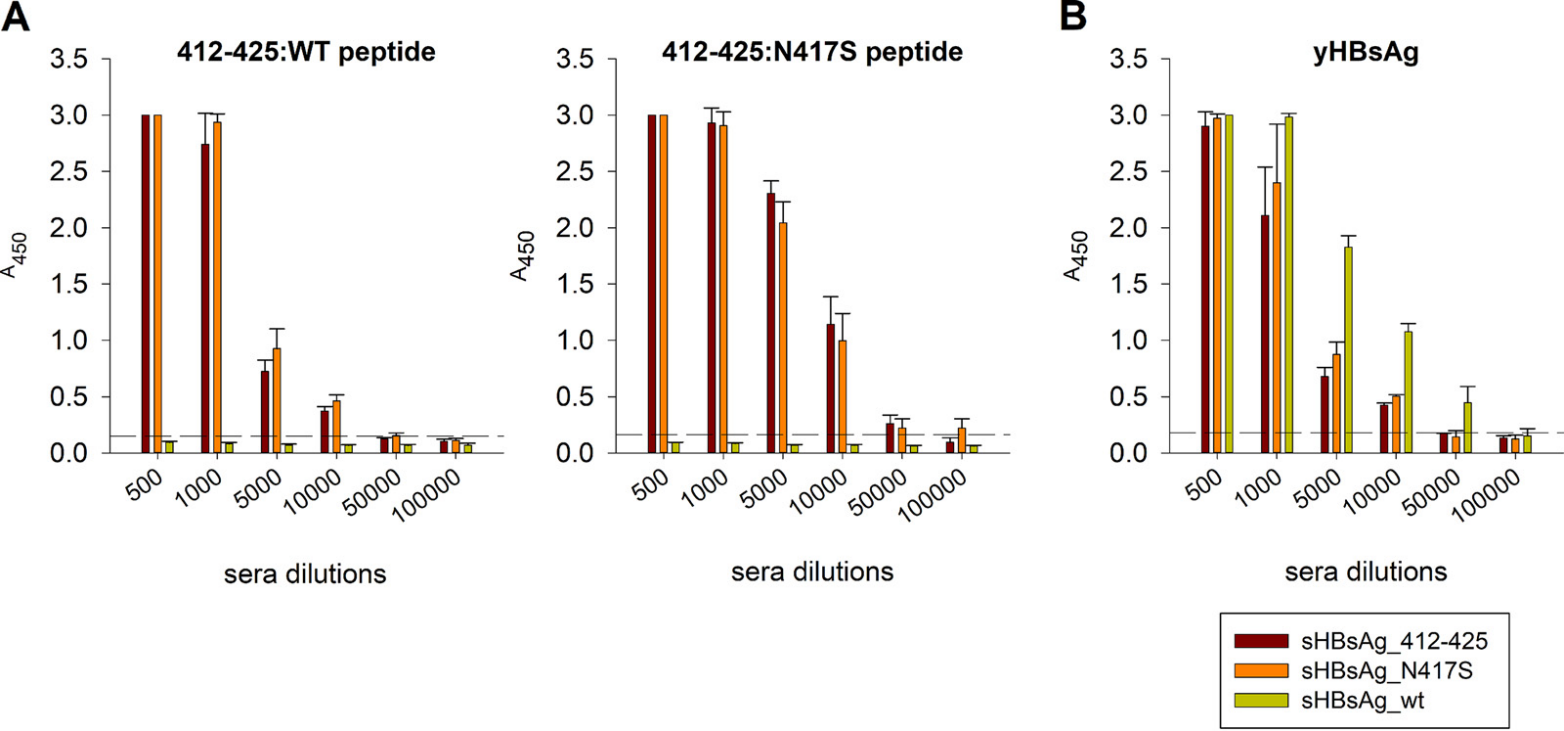
Analysis of antibodies elicited in mice following immunization with chimeric sHBsAg-based particles. (A) To analyze the binding of immune sera, streptavidin-coated microplates were coated with 5 μg/mL biotinylated synthetic peptides covering the HCV E2 412–425 and 412–425:N417S epitopes. (B) For analysis of the binding of immune sera to yeast-derived sHBsAg (yHBsAg), ELISA plates were coated with 5 μg/mL purified sHBsAg protein derived from P. pastoris. The dilution factors of the mouse sera are shown on the *x* axis. The mean *A*_450_ values are shown on the *y* axis. The background signal from the negative-control mouse sera was subtracted from the obtained results. The data represent the results from two independent experiments performed in duplicate, and error bars indicate standard deviations. The dashed horizontal line represents the cutoff value (three times the mean background value).

To investigate immunogenicity, 4 groups of 6 BALB/c mice were immunized subcutaneously with sHBsAg_412–425, sHBsAg_N417S, or wild-type sHBsAg (sHBsAg_wt) VLPs or phosphate-buffered saline (PBS). All mice were immunized using the squalene-based AddaVax adjuvant, an analog of MF59, which is licensed in Europe for human use. After mouse immunizations, we found that both sHBsAg_412–425 and sHBsAg_N417S VLPs elicited strong and specific antibody responses against biotinylated peptides corresponding to both wild-type E2 412–425 (412–425:WT) and E2 412–425 with the N417S change (412–425:N417S). Similar antibody titers of between 5 × 10^4^ and 10^5^ were observed for sera obtained after immunization with either of the constructs (Fig. 3A). These results showed that immunization with VLPs carrying E2 412–425 with or without the glycan shift could elicit an antibody response against the corresponding linear peptide regardless of the N417S change.

Detailed serum characterization showed that similar to the sHBsAg_412–425 VLPs, sHBsAg_N417S VLPs elicited an antibody response against purified yeast-derived sHBsAg protein (yHBsAg), with an antibody titer comparable to that of wild-type sHBsAg VLPs (Fig. 3B). These results confirmed that the insertion of E2 412–425 with or without N417 did not interfere with sHBsAg immunogenicity.

**FIG 4:**
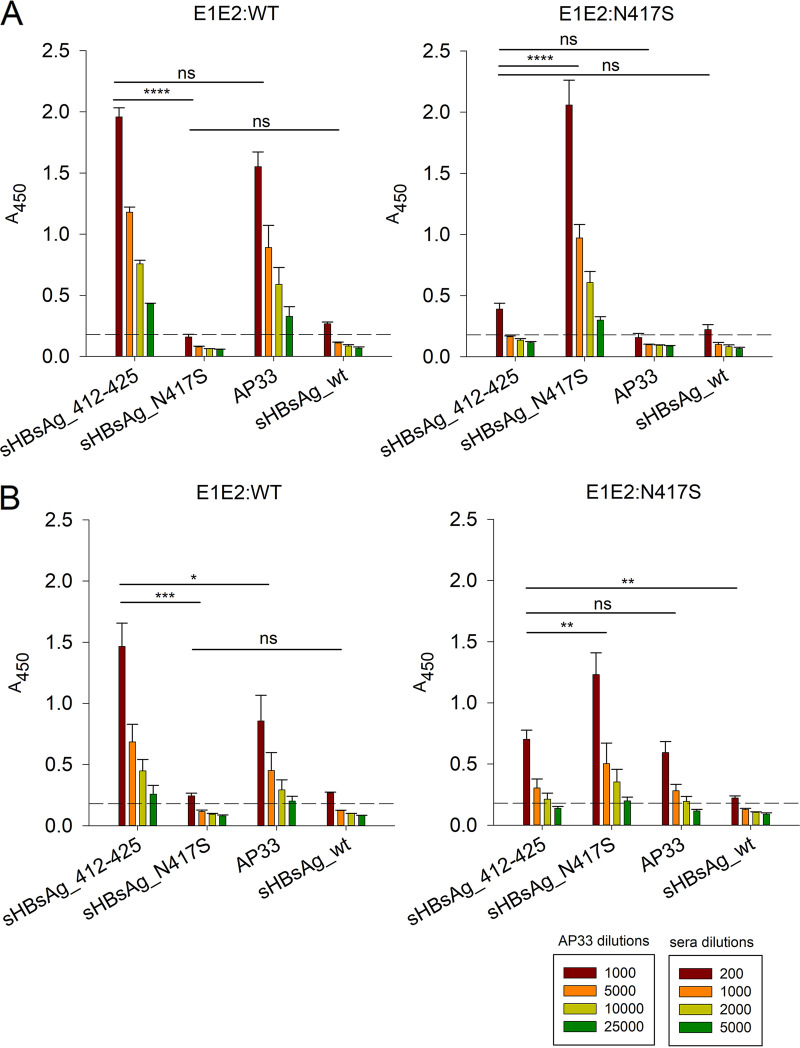
Reactivity of immune sera to E1E2 complexes with and without the N417S change expressed in mammalian cells. For the analysis of the binding of immune sera to E1E2 heterodimers expressed in mammalian cells, GNA-coated ELISA plates were incubated with native (A) or reduced/denatured (B) E1E2-containing cell lysates. The mean *A*_450_ values are shown on the *y* axis. Data were analyzed using two-way ANOVA (ns, *P* > 0.05; *, *P* < 0.05; **, *P* < 0.01; ***, *P* < 0.001; ****, *P* < 0.0001). Data represent the results from two independent experiments performed in duplicate, and error bars indicate standard deviations. The dashed horizontal line represents the cutoff value (three times the mean background value).

We next tested the reactivity of sera from the immunized mice against mammalian cell-expressed E1E2 complexes derived from genotype 1a (H77C) with or without the N417S change (E1E2:N417S and E1E2:WT, respectively) by ELISAs. Under native conditions (Fig. 4A), sera from mice immunized with sHBsAg_412–425 showed significantly reduced binding to E1E2:N417S in comparison to binding to E1E2:WT. This binding loss could be partially recovered by the incubation of sHBsAg_412–425 sera with denatured/reduced E1E2:N417S (Fig. 4B). A similar binding pattern was observed for AP33 (Fig. 4A and B). Sera from mice immunized with sHBsAg_N417S failed to recognize E1E2:WT but exhibited strong binding to E1E2:N417S under both native and denatured/reduced conditions (Fig. 4A and B). These results suggested that despite the reactivity of both sHBsAg_412–425 and sHBsAg_N417S sera to the synthetic linear peptides, their ability to recognize the full-length E1E2 glycoproteins was determined by the presence of the N417S change.

**FIG 5:**
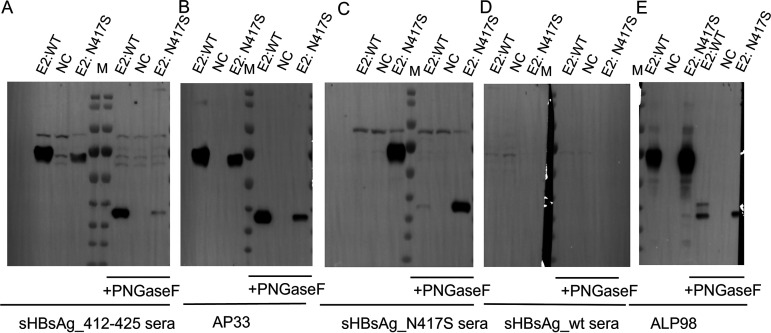
Reactivity of immune sera to E1E2 complexes treated with glycosidase. The E1E2 heterodimers with and without the N417S change were treated with endoglycosidase PNGase F. After overnight incubation, Western blotting under reducing conditions with mouse immune sera was performed (A and C). AP33 was used as a reference antibody (B), and sHBsAg_wt sera used as a negative control (D). ALP98, which binds to a different linear epitope on the E2 glycoprotein and was not affected by the N417S change, was used as a positive control (E). NC, negative control. Non-transfected HEK293 cell lysate is marked as NC. The protein ladder is marked with M, and the molecular weight in kDa is given.

To test the impact of the N-linked glycans on serum binding, we performed Western blotting with E1E2 complexes treated with PNGase F. sHBsAg_412–425 sera recognized glycosylated and nonglycosylated E1E2:WT and, with reduced affinity, E1E2:N417S (Fig. 5A). A similar binding pattern was observed for AP33 (Fig. 5B). For sHBsAg_N417S sera, we observed weak binding to E1E2:WT only when treated with PNGase F (Fig. 5C). Together, these data are in keeping with those shown in Fig. 4 showing sHBsAg_412–425 serum and AP33 binding to denatured/reduced E1E2:N417S, and they confirm that N-linked glycosylation at position N415 influences serum and mAb reactivities.

**FIG 6:**
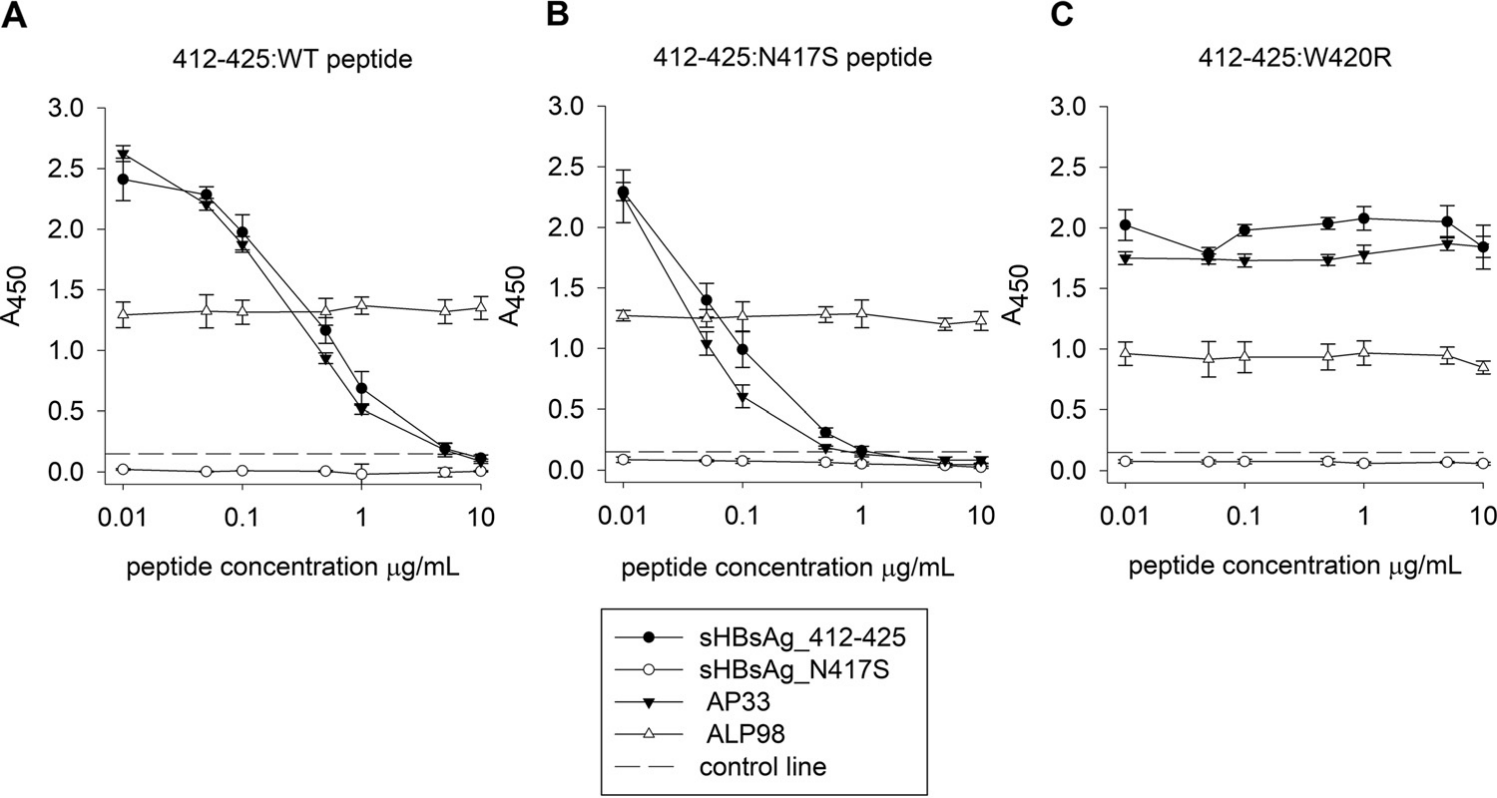
Immune serum binding to E1E2 complexes in a peptide competition ELISA. The sHBsAg_412–425 and sHBsAg_N417S sera and monoclonal antibodies AP33 and ALP98 were preincubated with serial dilutions of the peptide E2 epitopes 412–425:WT (A), 412–425:N417S (B), and 412–425:W420R (C). AP33 was used as a reference antibody, and ALP98 was used as a control. The antibody-peptide mix was then tested for reactivity against E1E2:WT by a GNA capture ELISA. The peptide concentrations are shown on the *x* axis. The mean *A*_450_ values are shown on the *y* axis. The data represent the results from two independent experiments performed in duplicate, and error bars indicate standard deviations. The dashed horizontal line represents the cutoff value (three times the mean background value).

Next, we performed a competitive ELISA in which mouse immune sera, AP33, and mAb ALP98 were preincubated with the 412–425:WT or 412–425:N417S peptide and transferred to microtiter plates coated with E1E2:WT (Fig. 6). We observed that the binding of sHBsAg_412–425 sera and AP33 to E1E2:WT was specifically inhibited by the 412–425:WT (Fig. 6A) and 412–425:N417S (Fig. 6B) peptides. Additionally, we performed a competitive ELISA with the 412–425:W420R peptide (Fig. 6C) in which W420, an essential contact residue for AP33 and CD81 receptor binding (28), was replaced by R. As expected, no inhibition of sHBsAg_412–425 sera and AP33 by the 412–425:W420R peptide was observed. Consistent with the data in Fig. 4 and 5, the sHBsAg_N417S sera failed to bind E1E2:WT. As expected, the peptides did not affect the binding of the control mAb ALP98, which recognizes a different linear epitope on the E2 glycoprotein (29), to E1E2:WT. When testing the inhibition of sHBsAg_N417S serum binding to E1E2:N417S (Fig. S3), we observed specific inhibition by both the 412–425:WT and 412–425:N417S peptides, and similar to the sHBsAg_412–425 serum, sHBsAg_N417S serum binding was not inhibited by the 412–425:W420R peptide. These results provided additional support for the similarities in binding patterns between the sHBsAg_412–425 sera and AP33.

**FIG 7:**
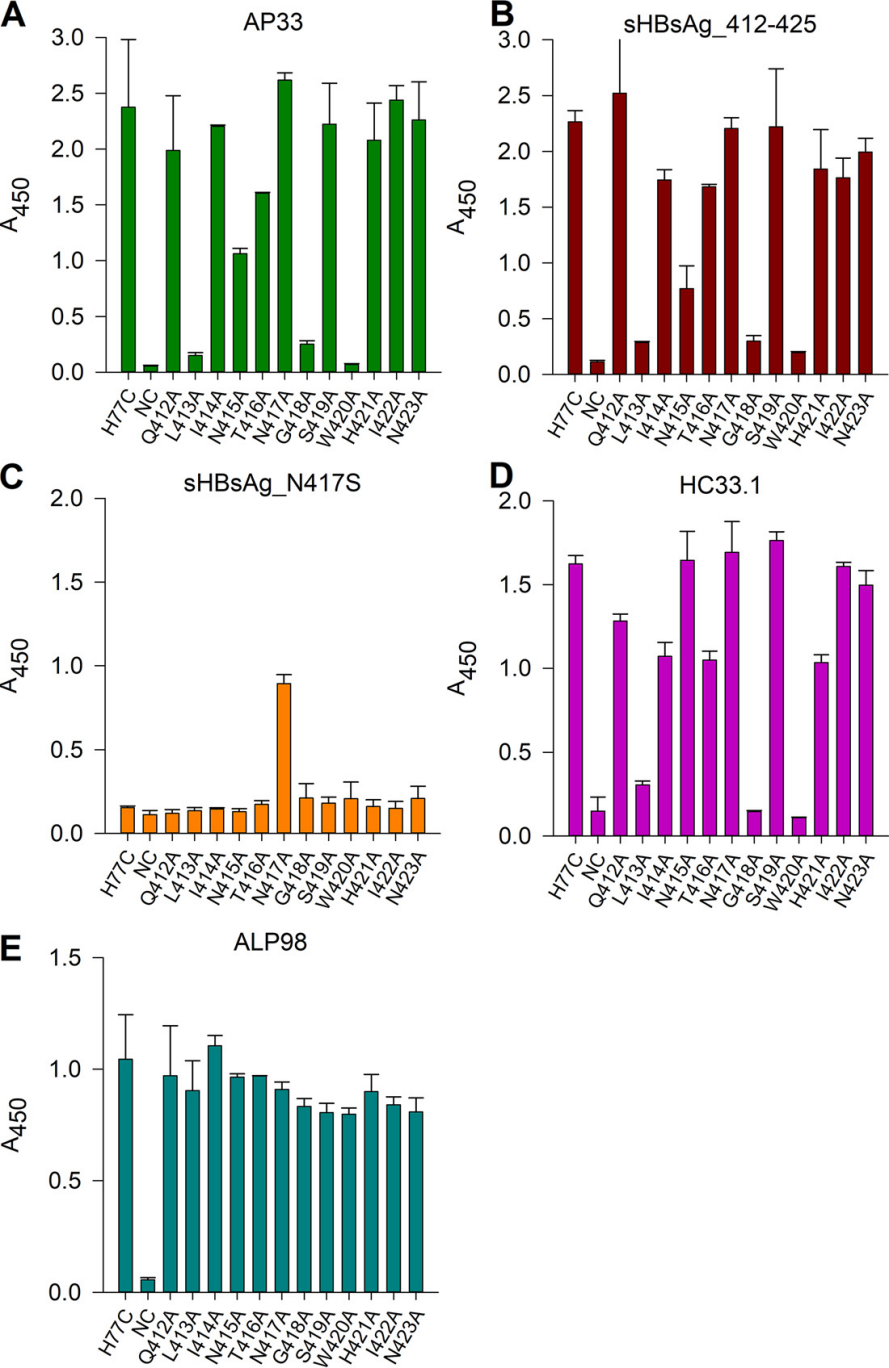
Reactivity of immune sera to a panel of E1E2 complex mutants in which residues across the 412–425 epitope were individually replaced by alanine. E1E2 heterodimer mutants were expressed in HEK293 cells, captured using GNA-coated ELISA plates, and probed with AP33 (diluted 1:2,500) (A), sHBsAg_412–425 and sHBsAg_N417S sera (diluted 1:500) (B and C), HC33.1 (2 μg/mL) (D) and ALP98 (diluted 1:1,000) (E). The mean *A*_450_ values are shown on the *y* axis. The substitution introduced into the E1E2 complex is annotated on the *x* axis according to the amino acid occurring within the H77C sequence, and the amino acid position is numbered relative to the H77C polyprotein chain. The data represent the results from two independent experiments performed in duplicate, and error bars indicate standard deviations.

To further compare sHBsAg_412–425 sera and AP33, we evaluated binding to a panel of E1E2 (genotype 1a H77C) mutants (Fig. 7) in which each residue across the 412–423 epitope was individually replaced by alanine. As shown previously (21, 30, 31), the binding of AP33 to E1E2 was abolished by alanine substitution of L413, G418, or W420 and reduced by alanine substitution of N415 (Fig. 7A). The binding profile of the sHBsAg_412–425 serum antibodies was similar to that of AP33, as their binding to E1E2 was abrogated by the same four mutations (Fig. 7B). This finding indicated that vaccination with 412–425_sHBsAg VLPs elicited AP33-like antibodies. As expected, sHBsAg_N417S serum was not reactive to the panel, except to E1E2 with the N417A change (Fig. 7C). For comparison, we performed an assay using mAb HC33.1. We observed that HC33.1 binding was hindered by alanine substitutions at L413, G418, and W420, which is in line with previously reported data (21, 30) (Fig. 7D). These results showed that antibodies elicited by sHBsAg_N417S VLPs have an E1E2 binding pattern different from those of AP33 and HC33.1. ALP98, which recognizes a different E2 epitope, was used as a positive control (Fig. 7E).

**FIG 8:**
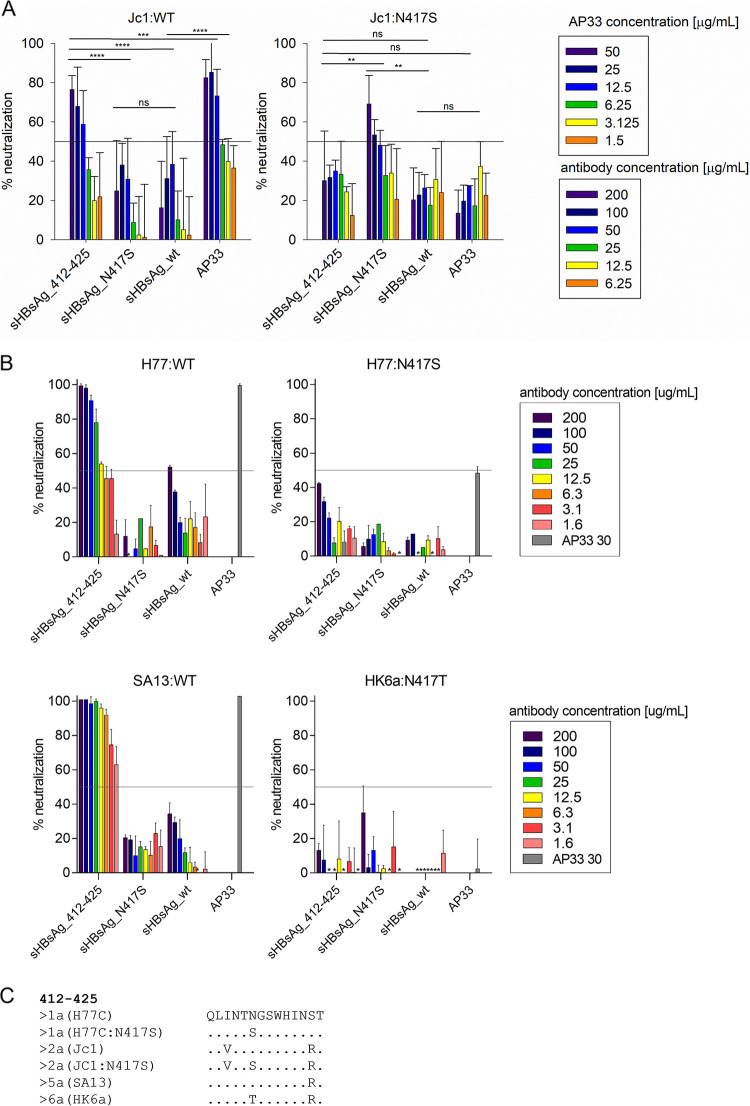
Chimeric VLP-induced antibodies neutralized HCVcc depending on the presence of the glycan shift change. Viruses were incubated with IgG purified from the sera of immunized mice and pooled for each experimental group. The neutralization effect (percent) was determined relative to cultures infected with the respective viruses in the absence of antibodies. (A) The antibodies were preincubated with wild-type HCV genotype 2a (Jc1:WT) or Jc1 with the N417S change (Jc1:N417S). The data represent the mean values from two independent experiments performed in duplicate, and error bars indicate standard deviations. Data were analyzed using two-way ANOVA (ns, *P* > 0.05; **, *P* < 0.01; ***, *P* < 0.001; ****, *P* < 0.0001). (B) The antibodies were preincubated with wild-type HCV genotype 1a (H77:WT), genotype 1a with the N417S change (H77:N417S), wild-type HCV genotype 5a (SA13:WT), or HCV genotype 6a with an N417T cell culture-adaptive change (HK6a:N417T). Each antibody concentration was evaluated in triplicate, and error bars indicate standard deviations. The solid horizontal line marks 50% virus neutralization. Neutralization values of ≤0% are not shown (indicated by *). (C) Amino acid alignment showing the sequence conservation of the region spanning aa 412 to 425. The sequence derived from HCV genotype 1a isolate H77C (GenBank accession no. AF011751) is used as the reference. Other sequences are designated according to genotype (isolate). Amino acids conserved relative to the H77C sequence are marked with dots.

Finally, we evaluated the neutralizing potency of IgG purified from sHBsAg_412–425 and sHBsAg_N417S mouse sera against HCVcc with or without the N417S/T change (Fig. 8). As previously reported, antibodies derived from mice immunized with sHBsAg_412–425 VLPs efficiently neutralized HCVcc lacking the glycan shift change, Jc1:WT (Fig. 8A), SA13:WT (16), and H77:WT (Fig. 8B), whereas no neutralization effect was observed for recombinants carrying an N417 change, Jc1:N417S (Fig. 8A), H77:N417S, and HK6a:N417T (Fig. 8B). Corresponding results were observed for AP33, with efficient neutralization of Jc1:WT, H77:WT, and SA13:WT; reduced potency against H77:N417S; and no neutralization of Jc1:N417S and HK6a:N417T. On the other hand, sHBsAg_N417S antibodies neutralized the Jc1:N417S virus carrying the N417S change to some extent at the highest IgG concentration tested (Fig. 8A); however, they did not show any neutralizing efficacy against H77:N417S and HK6a:N417T HCVcc (Fig. 8B). As expected, antibodies elicited by sHBsAg_N417S VLPs did not recognize viruses lacking the glycan shift change, Jc1:WT, H77:WT, or SA13:WT.

These findings confirmed that sHBsAg_N417S VLPs are immunogenic and elicit antibodies that specifically bind E1E2:N417S but appear to have limited neutralizing activity against HCVcc with the N417S glycan shift mutation, observed only in the context of certain virus isolates. Additionally, we described a high degree of similarity between AP33 and antibodies elicited by sHBsAg_412–425 VLPs.

## DISCUSSION

HCV E2 epitope I is a highly conserved, linear epitope targeted by several bnAbs and, thus, is an interesting target for rationally designed vaccines against this deadly pathogen. However, antibodies toward epitope I were reported to be found in only a low percentage of chronically infected individuals (15, 21). If epitope I is to be used as a future vaccine antigen, it should be efficiently presented to the immune system. Over the years, several broadly neutralizing monoclonal antibodies targeting this region have been reported in humans and animal models, including mouse AP33 (12, 13, 18), human HC33.1 (21), rat 3/11 (19), and human HCV1 (20). HCV1 was tested in the clinic as a potential HCV treatment in infected patients undergoing liver transplantation; however, several days after HCV1 infusion, patients experienced viral rebound, with the virus population showing escape variants consistently associated with the N417 change that cause the glycan shift escape mechanism (22, 23). Similar viral evasion was observed for *in vitro* selection with AP33, where prolonged incubation of HCVcc-infected cells with AP33 resulted in the occurrence of N417S/T changes, with N417S becoming dominant (17). Moreover, a previous analysis by Cowton et al. (31) via the HCV-GLUE database showed that N417S/T can be detected at a low frequency for the majority of HCV genotypes. These findings suggest that future vaccines targeting epitope I should be accompanied by antigens capable of overcoming N417-associated immune evasion mechanisms.

In this study, we designed sHBsAg-based VLPs presenting HCV E2 epitope I with the N417S change, shifting the N-glycosylation site from N417 to N415. We observed that despite the N415/N417 change, by ELISAs and immunogold staining, AP33 still bound the 412–425:N417S epitope presented on the surface of the sHBsAg-based VLPs with the same efficiency as that for the 412–425:WT epitope. This could be explained by the fact that the presentation of the 412–425:N417S epitope on the VLPs is conformationally different from its presentation in the context of the native E1E2 glycoprotein complex. The same AP33 binding was observed for sHBsAg_N417S VLPs expressed in HEK293F mammalian cells, which minimizes the possibility that AP33 binding is caused by changes in the Leishmania tarentolae glycosylation pattern.

After immunization with the AddaVax adjuvant, antibodies elicited by both sHBsAg_412–425 and sHBsAg_N417S VLPs showed dramatic differences in sensitivity to the E1E2 conformation and N-glycosylation status. sHBsAg_412–425 sera, like AP33, bound efficiently to E1E2 with the N1 glycosylation site at N417 (E1E2:WT) under both native and denatured/reduced conditions; however, they failed to recognize native E1E2:N417S. This binding could be partially restored by incubating sHBsAg_412–425 sera or AP33 with denatured/reduced E1E2:N417S, and this effect was not altered by additional deglycosylation. These results suggested that the lack of binding of sHBsAg_412–425 mouse sera to E1E2 with the N417S glycan shift change was due to neither the presence of the N-glycosylation site at N415 nor the amino acid change in epitope I but was most likely due to conformational hindrance.

Crystal structures of E2 revealed that the 412–423 epitope sequence shows high conformational flexibility, appearing disordered (32, 33), and may adopt several distinct conformations (34). Crystal structures of the 412–423 epitope peptide in complex with AP33 (35, 36) or HCV1 (37) indicated that epitope I folds as a short β-hairpin loop, and whereas the side chains of N417 and N423 point away from the antibody, N415 is buried in the antibody-binding groove (35–37). Therefore, the N-glycan shift from N417 to N415 would create untenable steric clashes with antibodies binding the β-hairpin conformation, abolishing antibody recognition. Considering the above-described results, it is possible that N417S causes a conformational change within the epitope I sequence, resulting in steric hindrance between antibodies and the glycan chain at position N415.

The other possibility is that the N417S change affects the global E1E2 conformation by limiting the accessibility of epitope I to AP33-like antibodies. In line with this hypothesis, previous studies have shown that amino acid changes within the 412–423 epitope or in the adjacent regions can result in global conformational changes in the HCV envelope heterodimer (38, 39). Similarly, global E1E2 conformation shifts were observed for HCVcc with selected N-glycans removed, resulting in neutralization sensitivity changes that could not simply be explained by the “glycan shield” model (40). Thus, N417S may induce a conformational change in native E1E2, which could lead to reduced epitope accessibility or recognition.

On the other hand, we showed that sHBsAg_N417S VLPs were capable of eliciting antibodies specifically recognizing E1E2 with the N417S change but not with the wild-type E2 sequence. mAb HC33.1, isolated from an HCV-infected blood donor, was previously developed and characterized (21, 27). HC33.1 can bind and neutralize E1E2 with a glycosylation site (N1) at N415 (i.e., E1E2:N417S) as well as with N1 at N417 albeit with lower sensitivity than E2 with the glycan shift change, suggesting that the N417/N415 glycosylation shift actually enhanced its binding (27). HC33.1 recognizes the 412–423 epitope in a semiextended β-sheet conformation, which allows N415 exposure to the solvent and, thus, does not hinder binding (30). In contrast, for sHBsAg_N417S sera, we observed binding to E1E2:N417S under both native and denatured/reduced conditions, but the sera showed no binding to native E1E2:WT. Additionally, unlike sHBsAg_412–425 serum binding to E1E2:N417S, the binding of the sHBsAg_N417S sera to E1E2:WT could not be restored by E1E2 denaturation. However, weak sHBsAg_N417S serum binding to E1E2:WT was restored by PNGase F treatment, which cleaves N-glycan chains at asparagine residues. Furthermore, in alanine scanning, sHBsAg_N417S sera efficiently recognized only E1E2 with the introduced N417A change. This change effectively removes the E2 N1 glycosylation site. For HC33.1, we observed a previously reported binding pattern, with a crucial role of residues L413, G418, and W420 (21, 30, 31). These results show that antibodies elicited by sHBsAg_N417S VLPs have an E1E2 binding pattern different from that of HC33.1. The recognition of E1E2:WT treated with glycosidase and the E1E2:N417A alanine mutant by sHBsAg_N417S sera suggests that the binding of the antibodies elicited by the sHBsAg_N417S VLPs is at least partially restricted by the presence of the N-glycan at N417, which is not the case for HC33.1.

Our results suggest that immunization with sHBsAg_412–425 VLPs elicits antibodies with an E1E2 binding pattern similar to that of AP33. The reactivity of the sera to E1E2 glycoproteins containing alanine changes across the 412–423 epitope showed that L413, N415, G418, and W420 were crucial binding residues for antibodies elicited by sHBsAg_412–425 and AP33 (12, 31, 36). Moreover, in peptide competition ELISAs, we showed that the binding of both sHBsAg_412–425 sera and AP33 to E1E2 was inhibited by 412–425:WT and 412–425:N417S but not by the 412–425:W420R peptide.

The results from the neutralization assay further emphasized the differences in sHBsAg_412–425 and sHBsAg_N417S serum binding to E1E2. As reported previously, antibodies elicited by sHBsAg_412–425 showed limited neutralization of isolates derived from genotype 2a (16). However, at high concentrations, antibodies purified from sHBsAg_412–425 sera successfully neutralized Jc1:WT HCVcc. The efficient neutralization of H77:WT HCVcc and the neutralization-sensitive SA13:WT HCVcc was further observed. As described previously for AP33 (13, 15, 26, 36), sHBsAg_412–425 sera did not neutralize H77, Jc1, or HK6a HCVcc carrying a glycan shift change. Although sHBsAg_N417S sera showed robust binding to E1E2:N417S, the antibodies appeared to be nonneutralizing or weakly neutralizing, depending on the specific strain of the analyzed HCVcc. sHBsAg_N417S antibodies showed a neutralizing effect against Jc1:N417S at high concentrations but failed to neutralize other variants carrying an N417 glycan shift change, H77:N417S and HK6a:N417T HCVcc. Comparing epitope I sequences of H77:N417S, Jc1:N417S, and HK6a:N417T, there are minor differences. Thus, it is possible that determinants either within or outside epitope I might influence the global E1E2 or epitope I conformation and antibody accessibility, resulting in differences in susceptibility to neutralization by sHBsAg_N417S-induced antibodies among the strains of the analyzed HCVcc.

As native epitope I presented in the hydrophilic loop of the sHBsAg-based VLPs efficiently induced bnAbs and epitope I with the N417 change induced E1E2:N417S-binding antibodies, future studies should focus on enhancing the titer and breadth of neutralizing antibodies elicited by antigens carrying epitope I with an N417 glycan shift change. This could be achieved by structure-based design of future antigens focusing on the stabilization of epitope I in the favorable conformation, resulting in neutralizing antibodies binding both wild-type epitope I and epitope I with the N417S change.

In this study, we have shown that antibodies elicited by sHBsAg-based VLPs presenting two variants of the 412–425 epitope targeted two distinct glycan variants of the HCV E1E2 heterodimer. Additionally, we observed striking similarities in the E2 glycoprotein binding patterns and HCVcc neutralization between sHBsAg_412–425 sera and AP33, suggesting that mouse immunization with sHBsAg_412–425 VLPs can elicit favorable broadly neutralizing AP33-like antibodies. Along with sequence variability and protein conformational flexibility, glycan shifting has been proposed as a mechanism of virus immune evasion (41–45), and the structural flexibility of the 412–423 epitope has been suggested as an immune evasion strategy (17, 30, 35–37, 46, 47). Constant changes allow HCV to escape binding by neutralizing antibodies or even to impede the induction of antibodies targeting epitope I by reducing its antigenicity. Additionally, the N417/N415 glycan shift contributes to immune evasion by inhibiting antibodies with affinity for the 412–423 epitope presented in the wild-type, β-hairpin conformation (30). The conformational flexibility of E1E2 and epitope I suggests that future epitope I-based vaccine antigens should elicit antibodies targeting more than one conformation and glycosylation variant of the 412–423 epitope.

## MATERIALS AND METHODS

### Plasmids.

Figure 1 summarizes the construction of the chimeric genes for sHBsAg_N417S particle generation. The region of the HCV E2 glycoprotein spanning residues 412 to 425 (isolate H77C [GenBank accession no. AF011751] [25]) with a single-point A1591G substitution resulting in the N417S change (numbering is according to isolate H77C) was inserted into HBV subtype adw2 sHBsAg (GenBank accession no. AF397207.1). Insertions of the HCV E2 epitopes into the hydrophilic loop of the sHBsAg protein were performed at position P127/A128. The construction of the chimeric genes coding for the sHBsAg_412–425 and sHBsAg_wt constructs was previously described (24).

The constructs were obtained by gene synthesis using L. tarentolae-adapted codons (Thermo Scientific). Synthesized genes were ligated into the BglII-NotI restriction sites in the pLEXSY_I-blecherry3 vector (Jena Bioscience).

For the expression of the HCV wild-type E1E2 glycoprotein in HEK293 cells for ELISAs, we used plasmids coding for full-length E1E2 derived from HCV genotype 1a (H77C) (25). For E1E2 with the N417S change, we introduced a single-point A1591G substitution into the sequence, coding for full-length E1E2, using a site-directed mutagenesis kit (QuikChange Lightning; Agilent). Mutagenesis accuracy was confirmed by sequencing.

Plasmids encoding alanine substitution mutants of genotype 1a strain H77C E1E2 were previously described (18).

### Leishmania tarentolae cultivation and protein expression.

The chimeric sHBsAg-based proteins were expressed using the inducible Lexsy expression system according to the manufacturer’s instructions (Jena Bioscience). Briefly, the plasmids were transfected into L. tarentolae cells by electroporation. The transfected cells were selected with bleomycin (100 μg/mL) in suspension culture. Subsequently, recombinant cell lines were cultivated in 25-cm^2^ tissue culture flasks filled with 10 mL of selective medium supplemented with hemin, protected from light at 26°C. T7 promoter-driven transcription was induced by the addition of tetracycline to a final concentration of 15 μg/mL. The cells were grown in agitated culture for 72 h at 26°C, aiming for a final optical density at 600 nm of 4 to 5.

### Cell lysis and ultracentrifugation.

Fifty milliliters of the tetracycline-treated cell culture was centrifuged at 4°C at 8,000 rpm for 15 min. The cell pellet was immediately resuspended in 5 mL of ice-cold lysis buffer (PBS buffer, 0.6% [vol/vol] Tween 20). The cells were sonicated, and the suspension was clarified by centrifugation at 4°C at 8,000 rpm for 35 min. The supernatant was left for 16 to 24 h at room temperature (RT) to form particles. Subsequently, the lysate was layered onto an OptiPrep (Sigma-Aldrich) gradient formed in ultraclear tubes (2 mL of 30% [vol/vol] OptiPrep, 2 mL of 24% [vol/vol] OptiPrep, 1.5 mL of 18% [vol/vol] OptiPrep, 1.5 mL of 12% [vol/vol] OptiPrep, and 1.5 mL of 6% [vol/vol] OptiPrep in PBS) and ultracentrifuged at 27,000 rpm for 16 h at 4°C. Next, 500-μL fractions were harvested and analyzed by Western blotting using anti-sHBsAg rabbit polyclonal antibodies (pAbs) (OriGene). The purity of the fractions was analyzed by SDS-PAGE with Coomassie R-250 staining. The fractions with the highest numbers of particles were pooled, and the protein concentration was measured by the Bradford assay (Bio-Rad). Finally, the OptiPrep solution was replaced with PBS using Amicon Ultra 100 kDa MWCO centrifugal units (Merck Millipore). These samples were used for further analysis and immunization.

### SDS-PAGE and Western blot analysis.

Analysis of the particle distribution after ultracentrifugation was carried out by SDS-PAGE using 4-to-12% gradient Bis-Tris gels in morpholineethanesulfonic acid (MES)-SDS running buffer. After electrophoresis, proteins were transferred onto a polyvinylidene difluoride (PVDF) membrane by electroblotting, and subsequently, the membranes were blocked overnight at 4°C with 3% nonfat milk in TBST (Tris-buffered saline, 0.1% [vol/vol] Tween 20). After blocking, the membranes were incubated for 1 h at RT with anti-sHBsAg pAb (OriGene) or mAb AP33 diluted in 0.3% nonfat milk in TBST, washed with TBST, and then incubated with secondary horseradish peroxidase (HRP)-conjugated antibodies (Santa Cruz Biotechnology). The results were developed using substrate for enhanced chemiluminescence (Thermo Scientific).

### ELISAs with VLPs.

To compare AP33 binding to sHBsAg_412–425 and sHBsAg_N417S VLPs by ELISAs, 96-well high-binding plates were preincubated with partially purified sHBsAg_412–425, sHBsAg_N41S, and sHBsAg_wt VLPs. Each well was then blocked with 3% (wt/vol) bovine serum albumin (BSA) in PBST (PBS buffer, 0.05% [vol/vol] Tween 20) for 2 h at RT. Primary mouse AP33, HC33.1, and ALP98 antibodies and anti-sHBsAg pAb were diluted in PBST with 0.3% (wt/vol) BSA, and serial dilutions were added to the wells. After washing, primary antibodies were detected with secondary HRP-conjugated antibodies (Santa Cruz Biotechnology). The reaction with the tetramethylbenzidine (TMB) substrate was stopped with 0.5 M H_2_SO_4_, and the signal intensity at 450 nm was measured using a plate reader.

### Electron microscopy.

For visualization of the particles, the OptiPrep gradient fractions were diluted 1:5 in PBS and deposited onto carbon-coated 200-mesh nickel grids. Negative staining was performed using 2% uranyl acetate. After staining, the samples were analyzed using a transmission electron microscope (University of Gdańsk, Poland). For immunogold labeling, sHBsAg_N417S particles deposited onto the grids were fixed using 1% (vol/vol) glutaraldehyde. Next, the grids were layered on top of AP33 antibodies diluted 1:40 in incubation buffer (PBS buffer, 0.1% [vol/vol] BSA-c (Aurion)) and incubated at 4°C for 16 h. After incubation with primary antibodies, the grids were washed six times using incubation buffer for 5 min at RT. Labeling was performed with goat anti-mouse IgG conjugated with 6-nm gold particles (Aurion), diluted 1:80 in incubation buffer, and left for 2 h at RT. After washing, the grids were stained with uranyl acetate, dried, and analyzed using a transmission electron microscope (University of Gdańsk, Poland).

### Immunization protocol.

Groups of 6 female BALB/c mice, 6 to 8 weeks of age, were immunized subcutaneously with a squalene-based oil-in-water nanoemulsion adjuvant (AddaVax; InvivoGen). The mice were immunized with 15 μg of protein on day 0 and 10 μg on days 14 and 28. The mice used as negative controls were immunized with the PBS-adjuvant mixture alone. All experiments on animals were conducted by an accredited company (Tri-City Academic Laboratory Animal Centre, Medical University of Gdańsk). The protocols were approved by the Local Committee on the Ethics of Animal Experiments of the University of Science and Technology in Bydgoszcz (permit no. 38/2018). All procedures were performed under isoflurane anesthesia, and all efforts were made to minimize suffering.

### Analysis of the antibody response by an ELISA.

Mouse sera were collected 2 weeks after the last immunization and pooled according to experimental groups. The antibody response against HCV E2 epitopes was measured by a direct solid-phase ELISA. Preblocked streptavidin-coated plates (Thermo Scientific) were incubated with biotinylated peptides at 5 μg/mL (GenScript) for 16 h at 4°C. After coating and washing, serially diluted mouse sera were added to the wells and incubated for 1 h at RT. Goat anti-mouse secondary HRP-conjugated antibodies (Santa Cruz Biotechnology) were used for detection. Similarly, the antibody response against sHBsAg protein was evaluated using ELISA plates coated with Pichia pastoris-derived sHBsAg protein at 5 μg/mL (OriGene). The plates were then blocked for 2 h with 3% (wt/vol) BSA in PBST, and serially diluted mouse sera were added to the wells as described above.

To test serum reactivity to E1E2 complexes, HEK293 cells were transfected with plasmids expressing wild-type E1E2 glycoproteins (E1E2:WT) and E1E2 glycoproteins with the N417S change (E1E2:N417S). Additionally, for the evaluation of serum binding to E1E2 alanine-scanning mutants, plasmids encoding alanine substitution mutants of genotype 1a strain H77C E1E2 were used to transfect HEK293 cells. Seventy-two hours after transfection, the cells were washed with PBS buffer and lysed in lysis buffer (PBS buffer, 0.5% Triton X-100). The clarified cell lysates were normalized against each other using mouse ALP98 and later used to perform ELISAs under denatured/reduced and native conditions. For the denatured ELISA, the HEK293 cell lysates were first diluted 50-fold in PBS buffer containing 50 mM dithiothreitol (DTT) and 2% SDS and incubated for 10 min at 100°C; for the native ELISA, the HEK293 cell lysates were diluted 50-fold in PBS alone. Next, the cell lysates were transferred to ELISA plates precoated with Galanthus nivalis lectin (GNA) and incubated for 16 h at 4°C. Next, the plates were blocked in PBST with 3% BSA and either used immediately or stored at −20°C. The pooled mouse sera were diluted in PBST containing 0.3% BSA. Finally, the binding of the antibodies to the recombinant proteins was detected by goat anti-mouse HRP-conjugated secondary antibodies diluted to 1:2,500 (Santa Cruz Biotechnology) and the TMB substrate.

### E1E2 PNGase F treatment.

The E1E2 heterodimers with or without N417 expressed in HEK293 cells were divided into two equal groups: one for digestion with PNGase F and one for the undigested control. The digestions were conducted for 16 h under native conditions at 37°C in the buffer provided by the manufacturer (New England BioLabs). The digested samples and the controls were separated by SDS-PAGE as described above and analyzed by Western blotting using mouse sera (diluted 1:1,000), AP33 (diluted 1:2,500), and ALP98 (diluted 1:1,500). Goat anti-mouse secondary HRP-conjugated antibodies (Santa Cruz Biotechnology) were used for detection. The results were developed using substrate for enhanced chemiluminescence (Thermo Scientific).

### Treatment of chimeric VLPs with PNGase F and endo Hf.

The sHBsAg-based VLPs were divided into two equal groups: one for digestion with PNGase F or endo Hf and one for the undigested control. The digestions were conducted as described above. The digested samples and the controls were separated by SDS-PAGE and analyzed by Western blotting using anti-sHBsAg pAb. Goat anti-rabbit secondary HRP-conjugated antibodies (Santa Cruz Biotechnology) were used for detection. The results were developed using substrate for enhanced chemiluminescence (Thermo Scientific).

### Peptide competition ELISA.

Peptides corresponding to wild-type epitope I (412–425:WT) or epitope I with the N417S (412–425:N417S) or the W420R (412–425:W420R) change, dissolved in PBS, were preincubated at concentrations of 10, 5, 1, 0.5, 0.1, 0.05 and 0.01 μg/mL with pooled mouse sera (1:500), AP33 (1:2,500), or APL98 (1:1,000) at RT for 1 h. The peptide-antibody mix was then tested for reactivity against GNA-captured E1E2:WT and E1E2:N417S by an ELISA, as described above.

### IgG purification from mouse sera.

IgGs were isolated from mouse sera using the NAb protein G spin kit (Thermo Scientific) according to the manufacturer’s instructions. Briefly, 300 μL of pooled mouse serum was transferred into spin columns and incubated for 30 min at RT. Subsequently, purified IgGs were eluted from the resin with low-pH elution buffer and concentrated using Amicon Ultra 100K centrifugal units (Merck Millipore).

### Virus stocks and neutralization assay for HCVcc genotype 2a.

To generate the Jc1:N417 mutant, the A1590G substitution resulting in the N417S change was introduced into the Jc1 E2 sequence (48) using the QuikChange Lightning site-directed mutagenesis kit (Agilent) and PCR primers introducing the mutation (Genomed). The sequence of Jc1:N417S was confirmed by E2 DNA sequencing (Genomed). HCVcc stocks were produced in Huh7.5 cells in Dulbecco’s modified Eagle’s medium (DMEM) supplemented with 10% fetal bovine serum (FBS) and penicillin-streptomycin (P/S).

The antibody neutralization assay was performed using Huh7-J20 cells, and virus infectivity levels were determined by a secreted alkaline phosphatase (SEAP) reporter assay, as described previously (49, 50). Briefly, Huh7-J20 cells were plated at a density of 2 × 10^4^ cells per well in a 96-well plate. In addition, ~250 focus-forming units (FFU) of virus were preincubated at 37°C for 1.5 h with serial dilutions of the tested antibodies prior to infection of cells. At 3h postinfection, the inoculum was replaced with fresh medium and incubated for 48 h. Infectivity was determined by measuring the SEAP activity released into the medium. Controls included noninfected cells and cells infected with the virus in the absence of the antibodies.

### Virus stocks and neutralization assay for HCVcc genotypes 1a, 5a, and 6a.

JFH1-based HCV recombinants with core-NS2 of the following genotypes (isolates) and the specified cell culture-adaptive changes were used: 1a (H77) with V787A and Q1247L (H77:WT) (51), 1a (H77) with the N417S change (H77:N417S) (39), 5a (SA13) with A1022G and K1119R (SA13:WT) (52), and 6a (HK6a) with F350S and N417T (HK6a:N417T) (53). HCVcc stocks were produced in Huh7.5 cells as described previously (53), and the envelope protein sequences were confirmed by Sanger sequencing. The cell-based *in vitro* HCV neutralization assay was performed as previously described (16, 52). Briefly, 7 × 10^3^ Huh7.5 cells per well were plated into a 96-well plate (Nunc) the day prior to infection. Virus-antibody mixes were prepared in a preplate. A volume of the virus stock resulting in counts of 50 to 180 FFU/well in pilot assays was added in a total volume of 7 μL per well and mixed with 3 μL of serially diluted purified IgG; for assays with H77:WT and H77:N417S, these volumes were 14 μL and 6 μL, respectively. For virus-only wells, medium was added instead of diluted purified IgG. The virus-antibody mixes were incubated for 1.5 h at 37°C with 5% CO_2_. After incubation, medium was added to a total volume of 40 μL per well. The total volume was transferred to the cell plate and incubated at 37°C with 5% CO_2_ for 4.5 h. Subsequently, the cells were washed with PBS, and fresh medium was added. Antibody dilutions were tested in triplicate. In each assay, at least six negative-control wells without virus and antibody and six virus-only wells were included. Cells were fixed with methanol after 48 h of incubation. After 10 min of incubation with 3% H_2_O_2_, the plate was incubated for 1 h with BSK (PBS containing 0.5% [wt/vol] BSA and 0.1% [wt/vol] skimmed milk). The plate was immunohistochemically stained with the primary antibody 9E10 (54) diluted 1:5,000 (in BSK) and ECL anti-mouse IgG HRP-linked whole secondary antibody (GE Healthcare-Amersham) diluted 1:500 (in BSK), both with incubation overnight at 4°C. FFU were visualized with a Bright-DAB solution kit (Immunologic) and counted automatically with an ImmunoSpot series 5 UV analyzer (CTL Europe GmbH) with customized software as previously described (55). The number of FFU in each well was related to the mean number of FFU in virus-only wells to calculate the percentage of neutralization.

### Data availability.

All relevant data that support the findings of this study are available from the corresponding author upon reasonable request.
