# Supplementary material for: Effect of Glycan Shift on Antibodies against Hepatitis C Virus E2 412–425 Epitope Elicited by Chimeric sHBsAg-Based Virus-Like Particles
Source: Microbiol Spectr. 2023 Jan 31;11(2):e02546-22. doi: 10.1128/spectrum.02546-22 (PMC10100762; doi:10.1128/spectrum.02546-22)
Supplement: Supplemental file 1 — Supplemental material. Download spectrum.02546-22-s0001.pdf, PDF file, 0.6 MB [file spectrum.02546-22-s0001.pdf]

Supplementary figures:

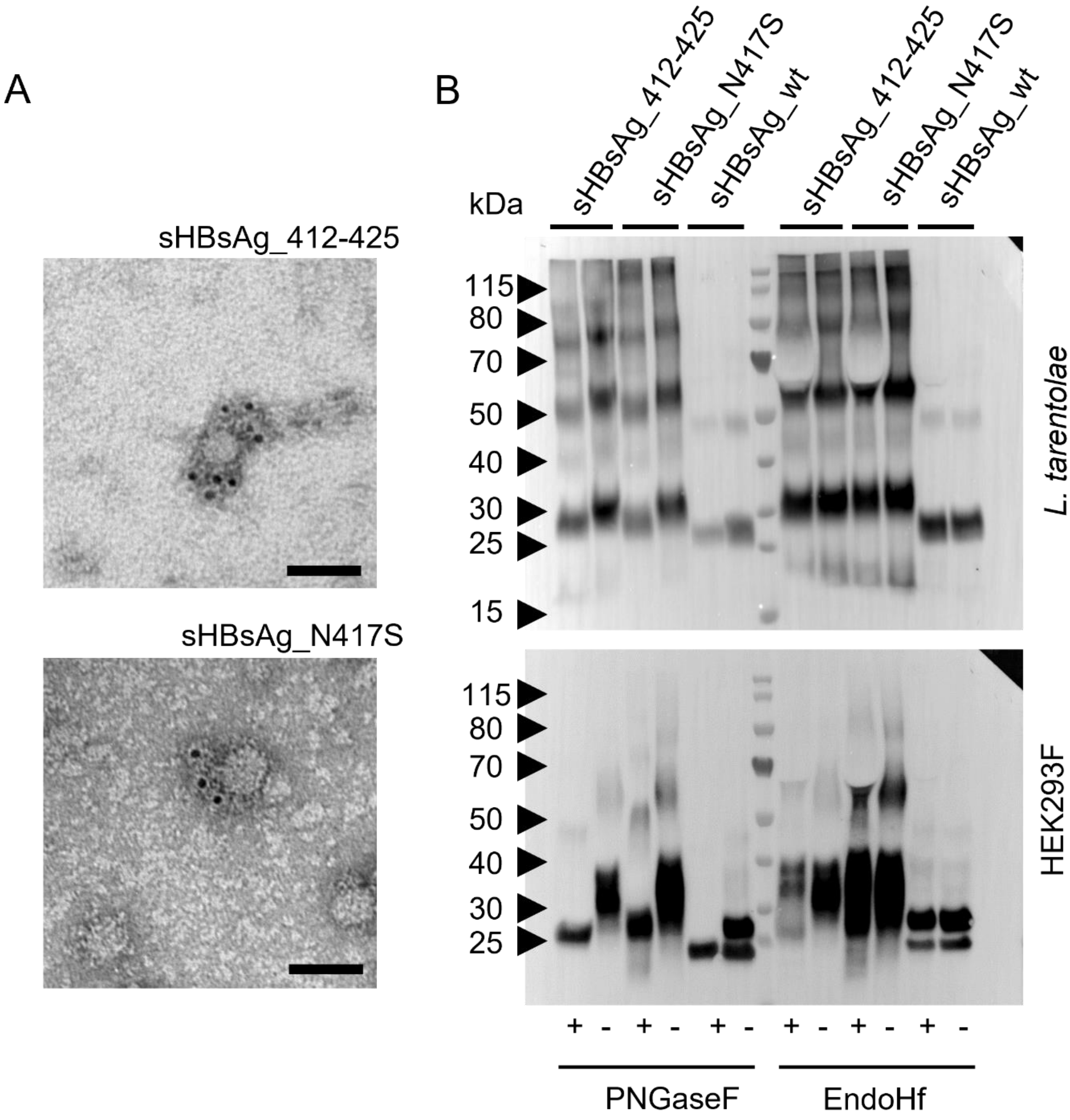

Fig. S1 (A) Immunogold labeling of the sHBsAg_412-425 and sHBsAg_N417S VLPs expressed in HEK293F cells with AP33. Secondary goat anti-mouse was conjugated with 6 nm gold particles. Scale bar: 50 nm. (B) Western blot analysis of the *L. tarentolae* and HEK293F-derived VLPs, treated with PNGaseF and EndoHf. pAb anti-sHBsAg were used for detection. On the left protein ladder, the molecular weight in kDa is given.

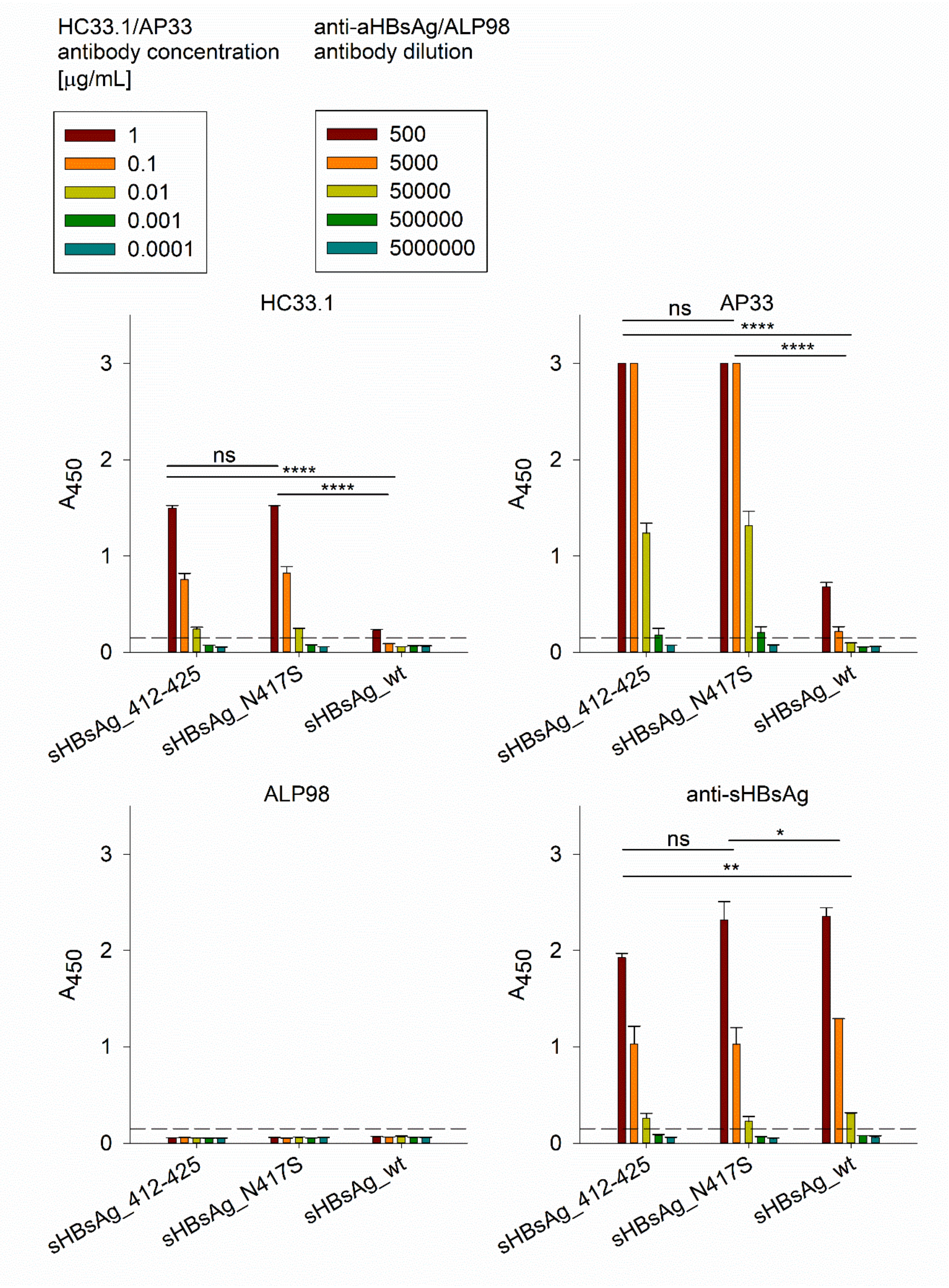

Fig. S2. Recognition of VLPs by a panel of antibodies. ELISA plates were coated with partially purified sHBsAg_412-425, sHBsAg_N417S and sHBsAg_wt VLPs as depicted on axis *x*. VLPs were probed with serial dilutions of epitope I specific AP33/HC33.1 and pAb anti-sHBsAg. mAb ALP98 was used as a negative control. Data was analyzed using two-way ANOVA (ns P

>0.05, *P<0.05, **P <0.01, ****P <0.0001). The mean A_{450} values are shown on axis *y*. The data represent the results from two independent experiments performed in duplicate, and error bars indicate standard deviations.

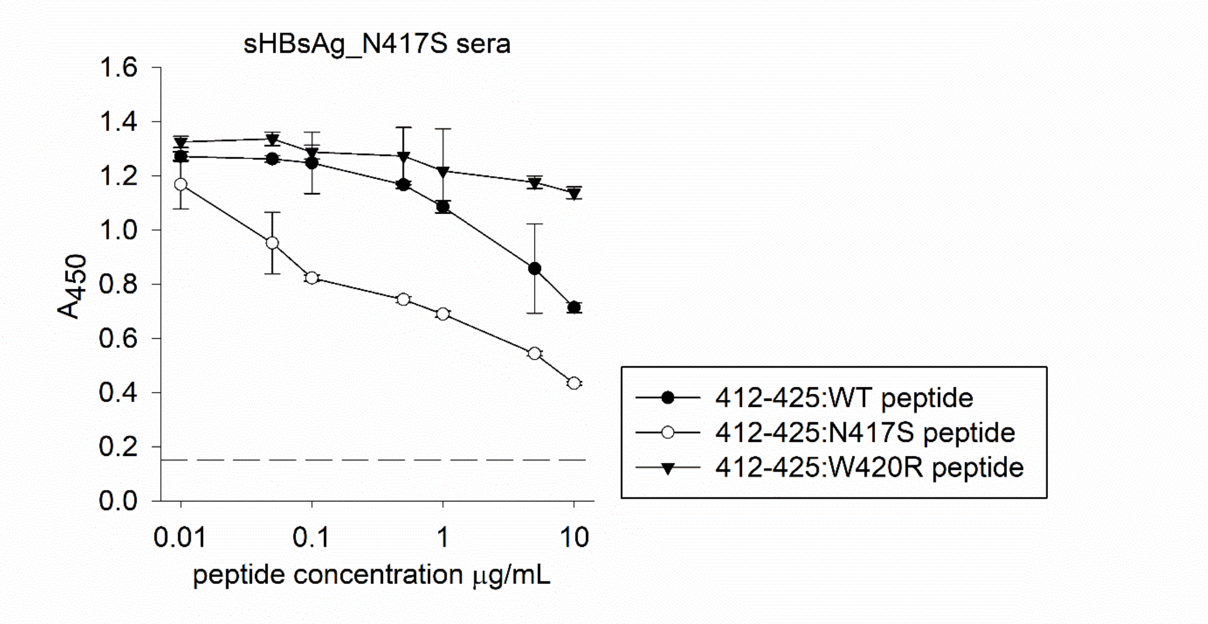

Fig. S3. sHBsAg_N417S sera binding to the E1E2:N417S complex in peptide competition ELISA. The sera sHBsAg_N417S sera was preincubated with serial dilutions of peptide E2 epitopes 412-425:WT, 412-425:N417S and 412-425:W420R. The sera/peptide mix was then tested for reactivity against E1E2:N417S in a GNA capture ELISA. The peptide concentration is shown on axis *x*. The mean A_{450} values are shown on axis *y*. The data represent the results from two independent experiments performed in duplicate, and error bars indicate standard deviations. The dashed horizontal line represents the cutoff value (three times the mean background value).
